# Prevention of alcohol consumption and transmission of human immunodeficiency virus: randomized clinical trial*

**DOI:** 10.1590/1518-8345.3393.3262

**Published:** 2020-05-11

**Authors:** Martha Dalila Mendez-Ruiz, Miguel Angel Villegas-Pantoja, Nohemí Selene Alarcón-Luna, Natalia Villegas, Rosina Cianelli, Nilda Peragallo-Montano

**Affiliations:** 1 Universidad Autónoma de Tamaulipas, Facultad de Enfermería de Nuevo Laredo, Nuevo Laredo, Tamaulipas, Mexico.; 2 University of Miami, School of Nursing and Health Studies, Coral Gables, Florida, United States of America.; 3 University of North Carolina, Chapel Hill School of Nursing, Chapel Hill, North Carolina, United States of America.

**Keywords:** Alcohol Drinking, Sexually Transmitted Diseases, Women, Young Adult, Randomized Controlled Trial, Nursing Care, Consumo de Bebidas Alcoólicas, Doenças Sexualmente Transmissíveis, Mulheres, Adulto Jovem, Ensaio Clínico Controlado Aleatório, Cuidados de Enfermagem, Consumo de Bebidas Alcohólicas, Enfermedades de Transmisión Sexual, Mujeres, Adulto Joven, Ensayo Clínico Controlado Aleatorio, Atención de Enfermería

## Abstract

**Objective:**

to know the effects of a nursing intervention to reduce alcohol use and risk factors for transmission of human immunodeficiency virus (HIV).

**Method:**

randomized single-blinded clinical trial performed by nurses with young women. The study included 66 participants in the intervention group and 66 participants in the control group. The instruments were the Alcohol Use Disorders Identification Test, the HIV Risk Behavior Knowledge and the Condom Use Self-efficacy Scale. Analysis of variance was used.

**Results:**

alcohol involvement decreased in the intervention group (F (1.119) = 50.28; p < 0.001; η^2^_p_ = 0.297), while HIV knowledge (F (1.130) = 34.34; p < 0.001; η^2^_p_ = 0.209) and condom use self-efficacy increased (F (1.129) = 27.20; p < 0.001; η^2^_p_ = 0.174). In addition, less participants consumed alcohol in the past week compared to the control group (χ^2^ = 15.95; p < 0.001).

**Conclusion:**

the nursing intervention had positive effects, which could help young women stay away from alcohol use and the risk of sexually transmitted infections. NCT: 02405481.

## Resumo

**Objetivo:**

conhecer os efeitos de uma intervenção de enfermagem para reduzir o uso de álcool e diminuir os fatores de risco para a transmissão do vírus da imunodeficiência humana (HIV, sigla em inglês).

**Método:**

ensaio clínico randomizado, simples-cego, realizado por enfermeiras com mulheres jovens. O estudo incluiu 66 participantes no grupo de intervenção e 66 participantes no grupo controle. Os instrumentos foram o Questionário de Identificação de Distúrbios de Uso de Álcool, a *HIVRisk Behavior Knowledge* e a Escala de Autoeficácia no Uso de Preservativos. Foi utilizada a análise de variância.

**Resultados:**

o consumo de álcool diminuiu no grupo de intervenção (F (1,119) = 50,28; p < 0,001; η^2^_p_ = 0,297), enquanto o conhecimento sobre o HIV (F (1,130) = 34,34; p < 0,001; η^2^_p_ = 0,209) e a autoeficácia no uso de preservativos aumentaram (F (1,129) = 27,20; p < 0,001; η^2^_p_ = 0,174). Além disso, menos participantes consumiram álcool na última semana em comparação com o grupo controle (χ^2^ = 15,95; p < 0,001).

**Conclusão:**

a intervenção de enfermagem teve efeitos positivos, que poderiam ajudar as mulheres jovens a ficarem longe do uso de álcool e do risco de infecção por doenças sexualmente transmissíveis. NCT: 02405481.

**Objetivo:**

conocer los efectos de una intervención de enfermería para reducir el uso de alcohol y disminuir factores de riesgo para transmisión del virus de inmunodeficiencia humana (HIV, siglas en inglés).

**Método:**

ensayo clínico aleatorizado, simple ciego, administrado por enfermeras a mujeres jóvenes. Participaron 66 personas en el grupo de intervención y 66 en el de control. Los instrumentos fueron el Cuestionario de Identificación de Trastornos debidos al Consumo de Alcohol, el HIV *Risk Behavior Knowledge* y la escala de Autoeficacia del Uso de Condón. Se utilizó análisis de la varianza.

**Resultados:**

en el grupo de intervención disminuyó el involucramiento con el alcohol (F (1,119) = 50,28; p < 0,001; η^2^_p_ = 0,297), aumentaron los conocimientos sobre el HIV (F (1,130) = 34,34; p < 0,001; η^2^_p_ = 0,209) y la autoeficacia para uso de condón (F (1,129) = 27,20; p < 0,001; η^2^_p_ = 0,174). También menos participantes bebieron alcohol en la última semana en comparación con el grupo de control (χ^2^ = 15,95; p < 0,001).

**Conclusión:**

la intervención de enfermería tuvo efectos positivos que podrían ayudar a las mujeres jóvenes a mantenerse alejadas del uso de alcohol y de contagio de enfermedades de transmisión sexual. NCT: 02405481.

## Introduction

Alcohol use is a global public health problem. Regions such as the Americas require special attention as it ranks second in alcohol consumption per capita^(^ 1 ^)^. However, it should be noted that the proportion of women who reported episodic alcohol use has tripled (drinking 4-5 drinks per occasion in the past 30 days), from 4.6% in 2005 to 13.0% in 2015^(^ 2 ^)^. Countries such as Mexico may have contributed to the increase in the prevalence of alcohol use, as there has recently been an increase in alcohol consumption among women aged 18 years or older. In this regard, it is estimated that between 2011 and 2016 the prevalence of excessive alcohol consumption (in the previous month) increased from 4.5% to 10.8% and daily consumption from 0.2% to 1.2% (in areas such as Tamaulipas, it is even higher, exceeding 1.5%)^(^ 3 ^)^.

Alcohol consumption is associated with multiple diseases and disorders, including mental problems, noncommunicable diseases such as liver cirrhosis, different types of cancer, cardiovascular diseases, trauma resulting from violence and traffic accidents^(^ 4 ^-^ 5 ^)^. Therefore, health professionals are also concerned about the increased susceptibility of women to the harmful effects of alcohol^(^ 6 ^)^. This is because the anatomical differences (higher percentage of adipose tissue than water) and metabolic differences (lower gastric activity of χ-alcohol-dehydrogenase, higher hepatic oxidation and lower effect of alcohol on gastric emptying), which allow them to quickly achieve higher blood alcohol levels in comparison with men^(^ 7 ^)^ and, consequently, be more exposed to the risks that this entails.

In this sense, the causal relationship between alcohol consumption and the incidence of serious diseases, such as human immunodeficiency virus (HIV) infection^(^ 8 ^-^ 9 ^)^ have been elucidated, which represents another serious public health challenge^(^ 10 ^)^. For example, it is known that alcohol consumption predicts sexual decision-making^(^ 11 ^)^, in addition to the fact that it could have negative effects on the cerebral prefrontal region^(^ 12 ^)^ - area associated with risk assessment during decision taking. That increases the likelihood of an individual having risky sexual behaviors, such as having impulsive sex behavior, having sex with multiple partners, and having sex without a condom. Such behaviors are associated with an increased chance to become infected with HIV^(^ 13 ^)^.

As proof of the complex but close relationship between alcohol use and the risks of HIV transmission, some Latin American studies present revealing data. For example, a study^(^ 14 ^)^ found that of those participants (young people from Northern Mexico) who reported having had sex, 41% had sex while under the influence of alcohol, and 61.2% without protection. They also highlighted that a higher alcohol consumption is associated with a higher prevalence of unprotected sex (r = 0.278, p < 0.01). Other researchers^(^ 15 ^)^ estimated that 30.3% of women in their study had sex while under the influence of alcohol, of which 47.4% had sexual contact with someone they just met. In addition, those who reported having had sexual contact while under the influence of alcohol had more than twice as many sexual partners (Mean = 4.3), compared to those who did not report having sex after taking any type of drug (Mean = 2.0; t = 6.37, p < 0.001). Findings such as those reported above deserve attention from the nursing staff, especially with regard to the development and implementation of interventions aimed at preventing alcohol use and HIV transmission in vulnerable groups such as women^(^ 13 ^,^ 16 ^)^.

Preventive interventions on this issue have shown variable efficacy, mainly because some have not demonstrated to have an impact on risk behaviors and they have only been limited to build knowledge^(^ 17 ^)^. In fact, nurses have developed few interventions by for the Mexican population. An example of evidence-based intervention is the program *Take care! Promote your health*, which was adapted and implemented in northern Mexico^(^ 18 ^)^ in order to delay the age of sexual onset and promote condom use. However, although it is effective, it does not include content on the use of alcohol and drugs and is addressed to parents and teenage children of both sexes - segment of the population different from the population at risk.

Considering the above, it is worth mentioning the work of a group of research nurses, who developed an intervention called Health, Education, Prevention and Self-Care (SEPA). It is an HIV prevention program developed for Latina women (including Mexican), initially residents in Chicago, United States of America^(^ 19 ^)^, but it has also been implemented in Hispanic women resident in Florida, United States of America^(^ 17 ^,^ 19 ^)^. SEPA effectively reduces the biological, behavioral and social risks that explain the transmission of HIV, including alcohol use. In fact, because of these attributes, it is listed by the Centers for Disease Prevention and Control as one of the few evidence-based nursing interventions recommended to be replicated^(^ 20 ^)^.

Because of its versatility, theoretical foundation and scientific evidence, SEPA is considered to have the characteristics of a program feasible to be implemented in Mexico. In addition, because it has been applied to young women, who are in the early stages of their sexual activity - within a context of personal, social and economic changes^(^ 21 ^)^ that could facilitate their involvement with alcohol - SEPA is an attractive opportunity for nursing practice. Especially in light of the insufficient scientific production, the demand for preventive strategies, as well as the wide distribution of nursing staff in primary health care. For this reason, an experimental study was proposed in collaboration with the SEPA group, the objective was to know the effects of a nursing intervention to reduce alcohol use and risk factors for transmission of human immunodeficiency virus (HIV). The following hypotheses were tested:

H_1_: women in the intervention group will be less likely to show involvement with alcohol compared to those in the control group.
H_2_: women in the intervention group will have a greater HIV knowledge than those in the control group.
H_3_: women in the intervention group will have an increased condom use self-efficacy than women in the control group.

## Method

The study was a randomized, single-blinded clinical trial with a control group, developed from January to July 2018 (NCT02405481). The participants were from two public universities in Tamaulipas, Mexico. Only women of Mexican nationality, aged between 18 and 30 years, with sexual activity in the past three months, availability to attend the intervention, and who signed an informed consent form were included.

Two research assistants conducted the recruitment two months before the intervention (by means of brochures and in person). In total, 543 applicants were received and examined. Of the applicants, 132 met the inclusion criteria and were randomly assigned to one of the two arms of the study: those who received SEPA were named as *intervention group* (IG; n = 66), while the *control group* (CG; n = 66) was composed of those who received a conventional preventive strategy. The sample size was sufficient to detect intra-subject and inter-subject differences, with a power greater than 80.0% and a medium effect size (ƒ = 0.25)^(^ 22 ^)^.

**Figure 1:**
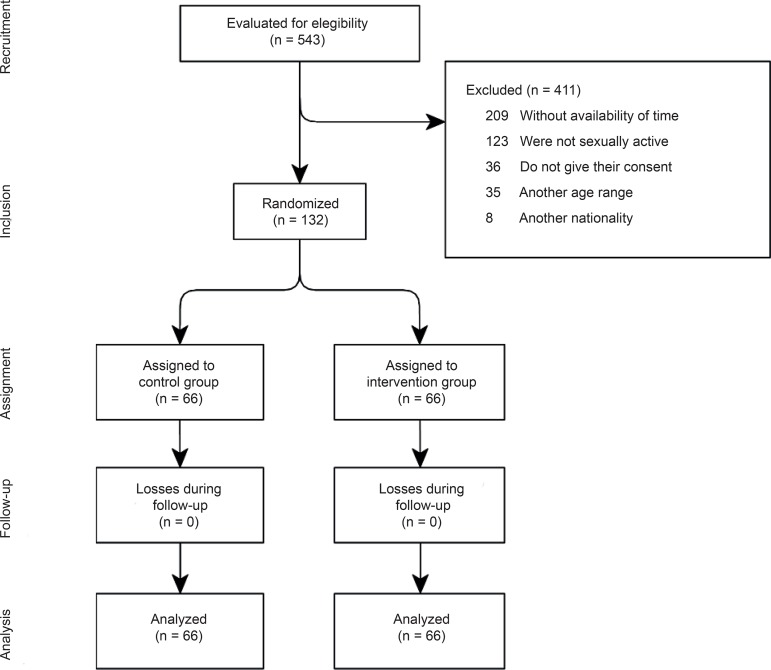
Research flow chart. Nuevo Laredo, Tamps, Mexico, 2018

Given that SEPA was not developed to be administered to large groups, 12 blocks were created with a maximum number of 11 participants each, six corresponding to the intervention group and six to the control group. The random assignment to the experimental and control groups was performed using an electronic spreadsheet with SAP function. The principal investigator, who did not know the identity of the participants, performed these procedures. This was a single-blinded masking study, since the participants did not know the group to which they were assigned. Figure 1 shows the diagram of the *Consolidated Standards of Reporting Trials* (CONSORT)^(^ 23 ^)^, with the flow of participants through the phases of the study.

SEPA intervention is based on the Social Cognitive Model of Behavior Change^(^ 24 ^)^. Based on the above, it aims that women identify their colleagues as models and listen to their experiences. Learning occurs by taking practical activities when observing live models (the facilitator and/or her group colleagues), by electronic means (such as images or awareness videos), when listening to instructions or reading printed materials^(^ 24 ^)^. It also encourages them to increase their knowledge through the interaction with their colleagues. According to these guidelines, the facilitator does not play the role of a teacher or try to impose her knowledge, but rather guides the dialogues and topics to be addressed. This encourages participants to discuss among themselves and carry out the activities of each meeting.

To maintain the reliability, prior training for facilitators was provided. This training lasted two months and was conducted by the principal investigator, who was trained by members of the research team of SEPA. Intervention manuals and presentations were used that helped to understand the contents and activities of each session. It is worth mentioning that one year before the intervention, manuals and contents were adapted to the local context by experts. For example, the epidemiological information on the Latino population in the United States of America was replaced by that of the Mexican population, as well as the language. Shortly after, a pilot study was conducted to examine the reliability of the instruments and identify areas for improvement. Based on the pilot study, it was concluded that the instruments showed acceptable reliability and no further adjustments were required.

The facilitators were nurses, with a level of education that ranged between bachelor’s degree and doctorate degree. Their responsibilities were a) to carry out SEPA sessions; b) to provide educational material, c) as well as to carry out strategies to retain participants during the intervention.

The research assistants carried out the recruitment two months before the beggining of the intervention. Dates for carrying out the interview were suggested to assess the inclusion criteria, which was conducted in private classrooms. Those who met the inclusion criteria were referred to the pre-test measurement and attended the intervention sessions. Conversely, those who did not meet the inclusion criteria were offered an educational lecture on a date other than the period in which SEPA was developed.

The study was carried out through five meetings: the pre-test and post-test measurements were performed in the meetings 1 and 5, respectively, while the meetings 2, 3 and 4 corresponded to the SEPA sessions. The sessions lasted 2.5 hours each, were administered at the rate of one session per week, and were taught in private classrooms within the institutions where the participants were. There were tables and chairs arranged in a semicircle to encourage interaction. The facilitators guided activities aimed at reducing alcohol involvement, avoiding sexually transmitted diseases and understanding the association between alcohol use and unsafe sex. Activities included sharing of printed information, role-plays, practical demonstration of skills and tasks to be developed in the community and at home.

The beginning of each session was aimed at reviewing the topics seen in the previous meeting, as well as discussing their tasks. At the end of each meeting, a moment was dedicated to feedback. Refreshments and raffles of gifts of $ 200 Mexican pesos (approximately $10 US dollars) were offered as a strategy to retain the participants. In addition, the facilitators maintained contact with the participants through text messages. By way of thanks, at the end of SEPA, certificates were provided to participants for having attended all the sessions.

The pre-test and post-test measurements in the control group were carried out in parallel to those in the intervention group. However, these participants were given a conventional educational session (lecture) that lasted 1 hour, offered by a facilitator. The lecture briefly approached the main topics of SEPA, such as the correct condom use and the risks of alcohol use. There was also a brochure with the same information, which was delivered at the end. A gift raffle was also carried out at the end of the talk.

The effectiveness of SEPA was measured in terms of its ability to modify three indicators: 1) decreased alcohol involvement (scores of the Alcohol Use Disorders Identification Test, AUDIT)^(^ 25 ^)^, 2) increased HIV knowledge (by means of the HIV Risk Behavior Knowledge)^(^ 26 ^)^, and 3) increased confidence for using condoms with their partner (Condom Use Self-efficacy Scale)^(^ 27 ^)^. These indicators were measured at the pre-test and post-test moments. Each instrument is described below.

A sociodemographic data card with 14 multiple-choice questions was included before the instruments. It aimed at collecting information such as age, marital status, labor aspects and use of alcoholic beverages (prevalence sometime in life, last year, last month and last seven days). The AUDIT test^(^ 25 ^)^ was used to identify the involvement with alcohol. This screening questionnaire, adapted to the Mexican population, is composed of 10 multiple-choice items that identify cases of excessive alcohol consumption. It consists of three domains: Itens 1 to 3 compose the risky alcohol use domain; itens 4 to 6 compose the alcohol dependence symptoms domain; and itens 7 to 10 belong to the harmful alcohol consumption domain. Together, they add up an overall score that ranges from 0 to 40, where a higher score indicates a higher involvement with alcohol. This instrument has adequate psychometric properties^(^ 28 ^)^.

The Spanish version of the HIV Risk Behavior Knowledge^(^ 26 ^)^ was used to measure HIV knowledge. This instrument is composed of 12 items, with four response options. However, the responses are analyzed through dichotomous choices (*True* or *False*). This instrument measures the knowledge about HIV transmission, prevention and consequences. The total score shows the number of items that were answered correctly (1 point for each correct answer) and, therefore, a score from 0 to 12 points can be obtained. High scores indicate a high level of HIV knowledge. This measurement has been previously used with a population mostly composed of Mexican women and has shown acceptable internal consistency (α = 0.75)^(^ 19 ^)^.

The Condom Use Self-efficacy Scale^(^ 27 ^)^ aimed to measure the effectiveness of women in negotiating condom use with their partner during sex. This instrument was developed and validated to be understood by the Latin American population, including Mexican women. It consists of 15 items with a semantic differential rating that are answered based on a general statement (*Please rate how confident you feel about doing what is mentioned in each of the following statements with your current partner*). The response scale ranges from 1 to 10, where *1 = Not confident at all*, *5 = Somewhat confident* and *10 = Very confident*. Thus, the overall score ranges from 15 to 150, where high scores suggest a high self-efficacy for condom use. Its internal consistency was considered as adequate (α = 0.92)^(^ 27 ^)^.

Descriptive statistics (percentages, measures of central tendency and measures of dispersion) and inferential statistics were used for the analysis of data. To support the research hypotheses, two-way analysis of variance (ANOVA) test (2 × 2) was used with repeated measures on one factor. The groups of belonging (IG vs. CG) was considered as an inter-subject factor and the intra-subject factor was the time of measurement (pre-test vs. post-test). This analysis identified the main effects of the group of belonging and those of the time of measurement, as well as those of the interaction between them.

Since the indicators of the variables of interest had no normal distribution, the transformation was done. Subsequently, the assumption of sphericity of a repeated-measures was verified by the Mauchly test (that is, if the matrix of variances-covariances is spherical; p > 0.05) and the assumption of homogeneity of variance by the Levene test (p > 0.05). In all cases, the assumptions were fulfilled, the *F-test* in the analysis of variance was interpreted and the partial eta-squared (η^2^_p_) was estimated. The *F*-value is an indicator of contrast of equality between the groups of study during the two measurement moments; the higher the value, the greater the probability of identifying differences. Regarding η^2^_p_, it quantifies the percentage of variance related to a main or interaction effect. In case of interaction between the factors, multiple comparisons (Bonferroni correction) were performed to compare the effects of the groups of belonging in the pre-test and post-test measurements in pairs.

In order to compare the sociodemographic characteristics between IG and CG in the baseline measurement, non-parametric inferential tests were used. In the case of continuous variables, the Mann-Whitney U test was used, while Pearson’s Chi-squared test (χ^2^) was used for dichotomous variables. The analyses were performed using SPSS v.22 for Mac OSX.

Regarding ethical aspects, the Research Ethics committee of the Nursing School of Nuevo Laredo, of the Autonomous University of Tamaulipas (protocol CA-A016) and the Public Education Secretary authorized the study through the Teacher Professional Development Program (protocol UAT-PTC-212). All procedures were in accordance with the General Health Law Regulation on Health Research in force in Mexico^(^ 29 ^)^, as well as the postulates of Declaration of Helsinki. In this way, the main ethical aspects considered were the search for the participants’ welfare and protection of their rights, minimization of damages, request for a written informed consent, anonymity, confidentiality of information, as well as freedom of participation.

## Results

The average age of participants was 20.02 years (standard deviation = 1.64) and they were aged between 18 and 29 years. Most of them were in a relationship, but were not married (53.8%), although a significant proportion was single (43.2%); few reported being married (3.0%). Tables 1 and 2 show some sociodemographic and alcohol use characteristics, segmented into control and intervention groups. It is noted that both groups started on equal terms because there were no significant differences neither in the continuous variables (Table 1) nor in the categorical variables (Table 2).

**Table 1:** Comparison of age and onset of alcohol use of the participants during the pre-test measurement. Nuevo Laredo, Tamps, Mexico, 2018

Variables	IG* (*n* = 66)	CG^†^ (*n* = 66)	U^‡^	*p*
	Mean (SD^§^)	Mean (SD^§^)		
Age in years	19.97 (1.41)	20.08 (1.85)	2115.00	0.767^||^
Age at onset of alcohol use	16.43 (1.55)	16.18 (1.90)	1675.00	0.413^||^

* IG = Intervention Group;

† CG = Control Group;

‡ U = Mann-Whitney U-Test results;

§ SD = Standard Deviation;

|| Statistical Significance (Mann-Whitney U-test)

**Table 2:** Prevalences of alcohol use and labor conditions of the participants during the pre-test measurement. Nuevo Laredo, Tamps, Mexico, 2018

Variables	IG* (*n* = 66) ƒ^§^(%^||^)	CG^†^ (*n* = 66) ƒ^§^(%^||^)	χ^2‡^	*p*
Alcohol use				
Sometime in life	60 (90.9)	61 (92.4)	0.099	0.753^¶^
Last year	51 (77.3)	52 (78.8)	0.044	0.833^¶^
Last month	34 (51.5)	32 (48.5)	0.121	0.728^¶^
Last seven days	17 (25.8)	16 (24.2)	0.040	0.841^¶^
Currently have a job	17 (25.8)	20 (30.3)	0.338	0.561^¶^

* IG = Intervention Group;

† CG = Control Group;

‡ χ^2^ = Pearson's Chi-squared Test results;

§ ƒ = Frequency;

|| % = Percentage;

¶ Statistical significance (Pearson's Chi-squared Test)

Regarding the involvement with alcohol, the first hypothesis proposed that, at the end of the intervention, women in the IG would have a reduction in the involvement with alcoholic beverages compared to those in the CG. When analyzing the AUDIT score using ANOVA tests, a significant main effect of the time of measurement was identified (F (1.119) = 61.48; p < 0.001; η^2^_p_ = 0.341), which means that there was a trend towards a reduction in the scores at the time of post-test. In addition, Table 3 shows a significant interaction between the group of belonging × time of measurement (F (1.119) = 50.28; p < 0.001; η^2^_p_ = 0.297), which suggests differences between the IG and CG scores, according to the time of measurement.

**Table 3:** Indicators of the effect of the intervention applied to young women. Nuevo Laredo, Tamps, Mexico, 2018

Indicators according to group	Pretest	Post test	(df*)F^†^	η^2^_p_^‡^	*p*
	Mean (SD^§^)	Mean (SD^§^)			
Scores in the intervention group					
Involvement with alcohol	1.84 (0.99)	1.24 (0.69)	(1.119) 50.28	0.297	0.001^||^
HIV knowledge^¶^	70.37 (31.00)	107.75 (28.67)	(1.130) 34.34	0.209	0.001^||^
Condom use self-efficacy	16840.98 (4116.47)	19803.93 (2500.29)	(1.129) 27.20	0.174	0.001^||^
Scores in the control group					
Involvement with Alcohol	1.73 (0.84)	1.70 (0.81)			
HIV knowledge^¶^	77.69 (36.70)	82.46 (32.90)			
Condom use self-efficacy	15741.70 (3860.59)	16632.75 (2991.43)			

* df = Degrees of freedom;

† F = Analysis of variance results;

‡ η^2^_p_ = Partial squared Eta;

§ SD = Standard Deviation;

|| Statistical significance of the interaction between time of measurement × group of belonging by two-way analysis of variance;

¶ HIV = Human Immunodeficiency Virus

**Figure 2:**
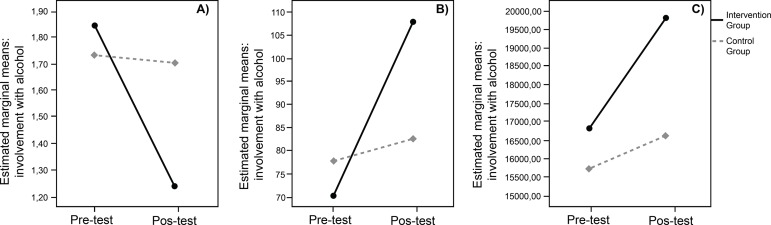
Graphs of the interaction between the group of belonging × time of measurement for the scores of A) involvement with alcohol, B) HIV risk behavior knowledge, and C) condom use self-efficacy in young women. Nuevo Laredo, Tamps, Mexico, 2018

Multiple comparisons were performed to corroborate this result and identify differences between IG and CG in each level of time of measurement. They confirmed that in the pre-test, the average in the IG (M = 1.84) showed no significant differences compared to that of the CG (M = 1.73; p = 0.517), whereas there were statistically differences in the post-test (Mean of IG = 1.24 vs. Mean of CG = 1.70; p < 0.001). These findings suggest that among women in the IG there was a decrease in the AUDIT scores after SEPA intervention, which indicates a reduction in the involvement with alcohol (Figure 2-A, graphically displays these trends).

On the other hand, as regards the women who consumed alcohol in the last seven days, a comparison between the proportions of alcohol consumers at the time of post-test was performed. Statistically significant differences were found, indicating that the percentage of consumers was lower in the IG (7.6%) than in the CG (36.4%, χ^2^ = 15.95, p < 0.001).

Regarding HIV knowledge, the second hypothesis proposed that, at the end of the intervention, women in the IG would have increased HIV knowledge scores. It was identified a significant main effect of time of measurement (F (1.130) = 57.40; p < 0.001; η^2^_p_ = 0.306), an effect with trend towards statistical significance in the group of belonging (F (1.130) = 3.33; p = 0.070; η^2^_p_ = 0.025), and a statistically significant interaction between the group of belonging × time of measurement (see Table 2; F (1.130) = 34.34; p < 0.001; η^2^_p_ = 0.209). These results suggest an increase in the of the post-test measurement scores, mainly among the participants in the IG. Multiple comparisons confirmed that there were no significant differences between the means of IG (M = 70.37) and CG (M = 77.69) scores in the pre-test (p = 0.218), but there was a significant difference in the post-test (Mean of IG = 107.5 vs. Mean of CG = 82.46; p < 0.001). As also shown in Figure 2B, the results reveal that the highest HIV knowledge scores were observed among those women who attended SEPA.

Finally, the third hypothesis proposed that, at the end of the intervention, women in the IG would have an increased condom use self-efficacy. In this section, significant main effects of the time of measurement (F (1.129) = 94.11; p < 0.001; η^2^_p_ = 0.422) and group of belonging (F (1.129) = 14.26; p < 0.001; η^2^_p_ = 0.100) were identified, as well as an interaction effect between the group of belonging × time of measurement (F (1.129) = 27.20; p < 0.001; η^2^_p_ = 0.174). This suggests that there was a significant increase in condom use self-efficacy scores among women in IG in the post-test (see Figure 2C). Multiple analyses showed that there were no statistically significant differences between IG (Mean = 16840.98) and CG (Mean = 15741.70) in the pre-test (p = 0.117), but there were in the post-test (Mean of IG = 19803.93 vs. Mean of CG = 16632.75, p < 0.001).

## Discussion

The findings of this experimental study revelead that the preventive intervention SEPA had positive effects in reducing alcohol consumption and preventing some risk factors for HIV transmission among young Mexican women. This provides scientific evidence on the effectiveness of SEPA intervention, which has been applied to Latino women from different contexts^(^ 17 ^,^ 19 ^)^, and no similar intervention had been applied within Mexico. It also shows that nurses can improve their actions (in this case, primary health care nurses) by their own means in order to improve population health.

Regarding the first study hypothesis, it was found that compared to the control group, women who attended the SEPA had a significant decrease in AUDIT questionnaire scores, as well as in alcohol consumption in the last seven days (from 25,8% in the pre-test to 7.6% in the post-test). These results are in line with those reported in a study of 548 Hispanic women living in the US, which has shown that after attending the SEPA, the frequency in which participants reported having been alcoholized decreased^(^ 17 ^)^. In comparison, our results also show an improvement in terms of AUDIT score.

The use of AUDIT can be an advantage, as it is a reliable instrument, whose scoring not only gives an idea of the amount and frequency of alcohol consumption, but also represents an approach on the possible consequences of alcohol consumption, as well as the symptoms of alcohol abuse and alcohol dependence^(^ 25 ^)^. It has also been reported that the scores are associated with the attitudes and reasons why the individual drinks^(^ 30 ^)^, so that it represents the relationship that the individual has with alcohol in a more comprehensive way. In this sense, the use of screening instruments such as AUDIT constitutes an advantage for the nursing practice: they allow prioritizing care and resources, specifically for the most vulnerable individuals. Therefore, such actions could also have benefits - economic and social - for the health system.

Regarding the second hypothesis, at the end of the intervention, it was found that in IG there was a significant increase in HIV knowledge. These findings are in line with those reported in other studies^(^ 17 ^,^ 19 ^)^, in which SEPA was applied, with a significant increase in HIV knowledge scores being observed from the third month of follow-up. Our results are also in line with an experimental study conducted in Cuba^(^ 31 ^)^, with a similar methodology (intervention using audiovisual material, reflection, group talks, debates, information displayed in slides and educational material), which lasted six months. This research reported an increase in HIV and AIDS knowledge of young participants at the end of the intervention.

Increases in HIV knowledge can be beneficial, since it is associated with increased condom use^(^ 32 ^-^ 33 ^)^, less risky sexual behavior^(^ 34 ^)^, greater perception of HIV risks^(^ 35 ^)^, and less stigmatization towards individuals living with HIV^(^ 36 ^)^. In addition, since there are still prejudices in the Mexican population when talking about this disease^(^ 37 ^)^, the contribution of SEPA could be the changes in attitudes towards HIV and in HIV knowledge. This latter aspect is important for the nursing staff, since the lack of objective HIV knowledge has been pointed out, as well as the persistence of misconceptions concerning HIV prevention and treatment^(^ 38 ^)^. Primary health care nurses could address many of these prejudices.

Finally, regarding the third hypothesis, there was a significant increase in condom use self-efficacy among women who attended SEPA. This is in line with studies that report positive effects in terms of increased condom use self-efficacy^(^ 17 ^,^ 19 ^,^ 32 ^)^. Self-efficacy is defined as a personal perception of the ability to perform an action^(^ 24 ^)^, in this case, condom use when having sex with a partner. The reason why nursing should promote self-efficacy by means of practical activities is that when people feel capable of using a female or male condom, they are more likely to use it^(^ 39 ^-^ 40 ^)^.

It is worth mentioning that the nursing staff in primary health care units frequently carries out demonstrative activities of the use condom; however, in few occasions they are carried out in non-hospital settings as shown here. For this reason, it is considered that SEPA could be a means of connecting nurses even more with the population in a community setting.

Among the limitations of this research, it is worth noting that there are no follow-up data supporting the persistence of the changes achieved beyond the end of the intervention. On the other hand, given the nature of the self-report instruments, it is possible that when discussing delicate issues (such as the use of alcohol and drugs), the mood of the participant and the context may have an impact on the filling^(^ 41 ^)^. Finally, a limitation for the generalization of the results is the selection of the sample.

Since this study is one of the first approaches carried out in Mexico, its implementation was developed in an educational environment, and participants were young women with higher education and wide availability to attend the intervention during the study period. It is possible that in the general population the efficacy and understanding of the intervention will vary. In particular, some aspects could be adapted to a real context.

For example, carrying out an intensive recruitment, as it was performed here (to maintain several intervention groups simultaneously), could be a challenge in everyday conditions. Alternatively, a sequential recruitment could be carried out and, thus to form intervention groups throughout the year. This could also contribute to include part of the eligible population that was excluded due to lack of time. In future research, it will be necessary to consider these aspects when applying SEPA in a community context that faithfully represents other vulnerable segments of the Mexican population.

## Conclusion

Based on the post-test measurements, it can be concluded that SEPA has the potential to reduce the involvement with alcoholic beverages and certain risk fators for HIV transmission in Mexican women. It has also proved to be understandable to the participants and to the nursing staff who administered it. It is noteworthy that it showed better results than a conventional strategy used by the nursing staff in a community context. Due to its methodological characteristics and results, it is considered as a strategy that could be incorporated into the preventive actions performed by the nursing staff with young women. This could benefit primary health care nurses, because in the future SEPA could represent a preventive strategy based on evidence with a positive impact on the public health of the region. However, further studies should be conducted to confirm its usefulness in people of different educational levels.
